# Supplementary material for: Spatially and temporally precise microbiome profiling in the small intestine using the SIMBA capsule with X-ray tracking
Source: Front Microbiomes. 2024 Jul 9;3:1321624. doi: 10.3389/frmbi.2024.1321624 (PMC12993646; doi:10.3389/frmbi.2024.1321624)
Supplement: Supplementary file 1 [file DataSheet_1.docx]

Supplementary Material


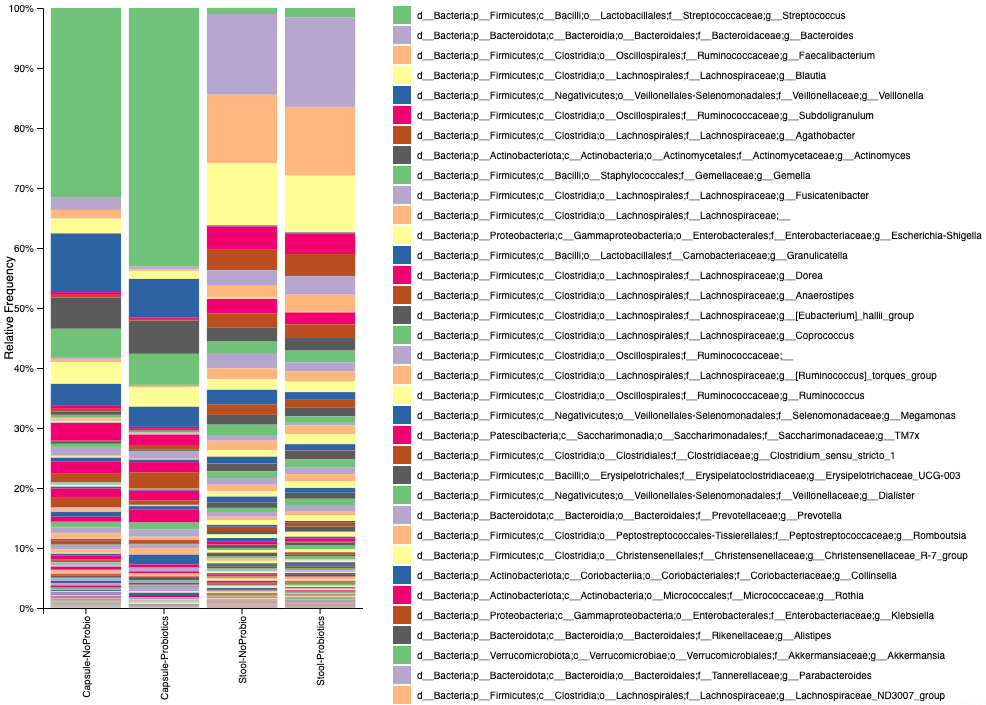


**Supplementary Figure 1.** Grouped taxonomic bar plots showing genus level microbiome composition after removal of the *Lactobacilli* and *Bifidobacteria* from all groups.

**
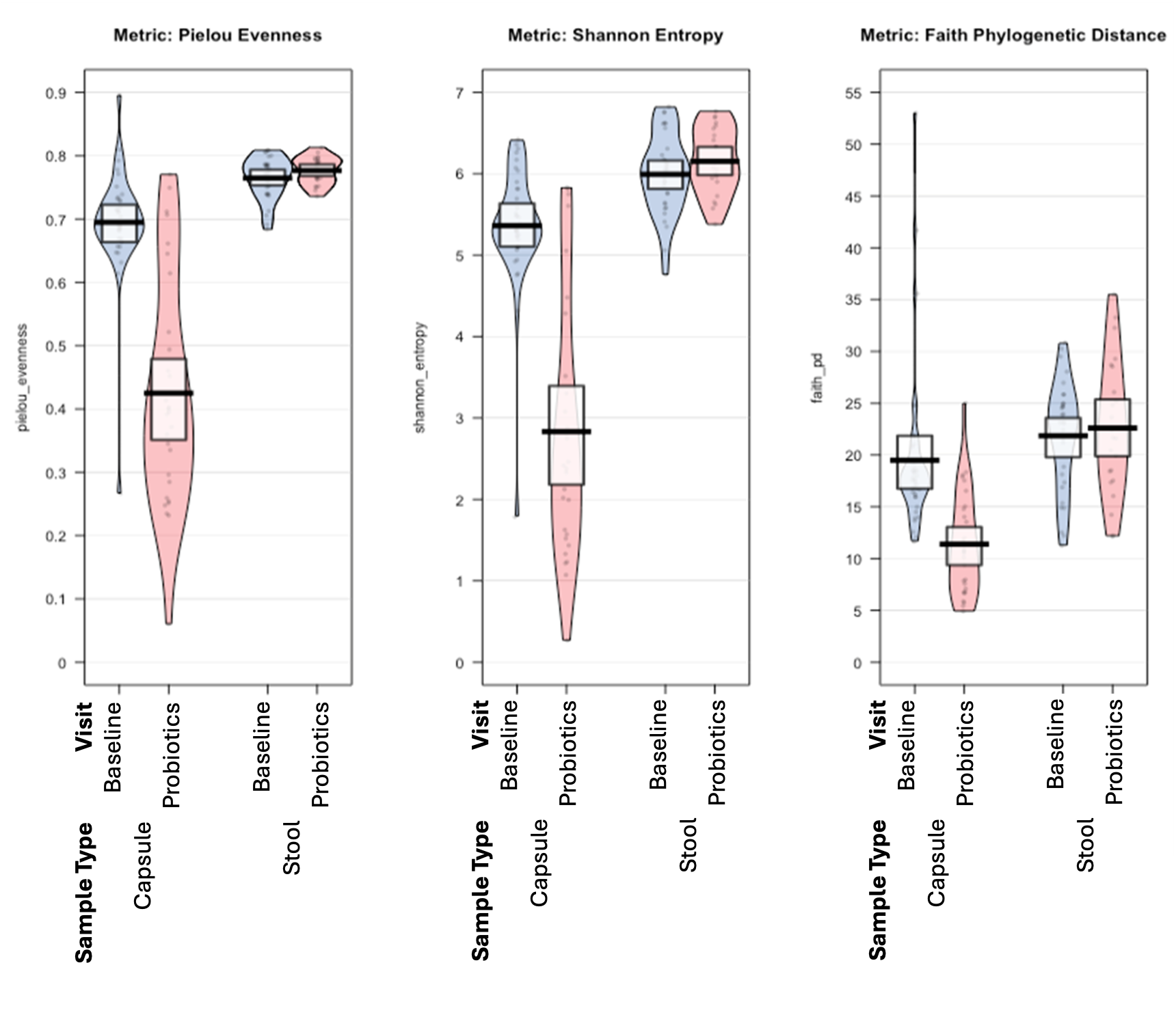
**

**Supplementary Figure 2.** Alpha diversity metrics.


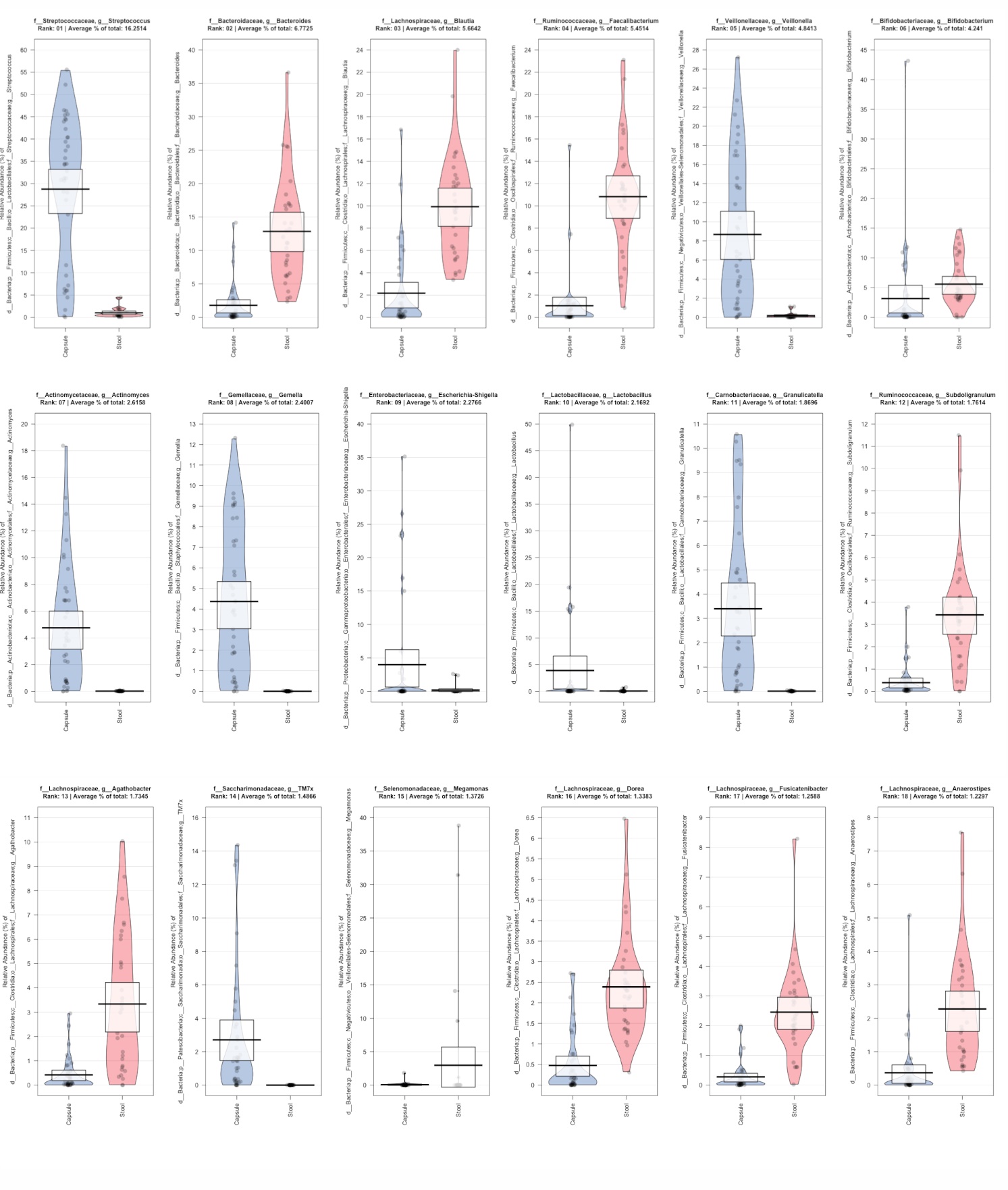


**Supplementary Figure 3.** Top 18 classifiers of the comparison between baseline capsule and stool samples.
